# Assessment of Relapse in Patients with Schizophrenia during COVID-19 pandemic

**DOI:** 10.1192/j.eurpsy.2022.790

**Published:** 2022-09-01

**Authors:** E. Abdelfattah, M. Bassiony, M. Sehlo, A. Zayed, S. Atwa

**Affiliations:** 1 Faculty of medicine, Zagazig University, Psychiatry, Zagazig, Egypt; 2 Faculty of medicine Zagazig university, Psychiatry, Zagazig, Egypt

**Keywords:** compliance, Covid-19, Relapse, schizophrénia

## Abstract

**Introduction:**

People with schizophrenia could be more substantially influenced by the emotional stress brought on by the COVID-19 pandemic, resulting in relapses or worsening of an already existing mental health condition because of the high susceptibility to stress.

**Objectives:**

This study aimed to assess the presence of relapse and its risk factors among patients with schizophrenia during the era of COVID 19 pandemic.

**Methods:**

This study included 90 adults who met DSM-5 criteria for schizophrenia and were diagnosed by (SCID-I) Arabic version and who are following up at the outpatient psychiatric clinic, Zagazig University Hospital, Egypt, Positive and Negative Syndrome Scale (PANSS) measuring severity of symptoms, Compliance Rating Scale (CRS) and World Health Organization Disability Assessment Schedule 2.0 (WHODAS 2.0). scales were conducted on those patients before COVID-19 pandemic from January to April 2019 and repeated on September to November 2020 during COVID 19 pandemic to compare clinical parameters between those two periods as to detect any deterioration in their clinical state.

**Results:**

The mean score of compliance rating scale (CRS) was decreased after COVID-19 pandemic compared to before COVID 19 (P<0.001).The mean score of PANSS scale positive subscale (P) had increased after COVID-19 pandemic compared to before COVID 19 (P<0.001).

**Conclusions:**

There was a deterioration of the clinical state of schizophrenic patients during COVID-19 pandemic especially the positive symptoms and following up the news about COVID-19 regularly, decreased level of family support, lower level of compliance with treatment, and having a family member with COVID-19 infection or death were the risk factors for relapse.

**Disclosure:**

No significant relationships.

